# NIR-responsive CN-Pt-GEM hydrogel induces necroptosis and immunotherapeutic responses prevent postoperative recurrence and wound infection in lung carcinoma

**DOI:** 10.1186/s12951-024-02568-4

**Published:** 2024-06-21

**Authors:** Meng Wang, Rui Cai, Zhiwu Zhang, Longbao Feng, Ziying Lei, Fengpin Wang, Zhongjian Yu, Lu Liu, Xia Yang, Huili Guo, Bingjie Shan, Shiting Xu, Rui Guo, Shuzhong Cui, Yanfang Zheng

**Affiliations:** 1 Affiliated Cancer Hospital and Institute of Guangzhou Medical University, Guangzhou, 510095 China; 2 State Key Laboratory of Respiratory Disease, Guangzhou, China; 3 Key Laboratory of Biomaterials of Guangdong Higher Education Institutes, Guangdong Provincial Engineering and Technological Research Center for Drug Carrier Development, Department of Biomedical Engineering, Jinan University, Guangzhou, 510632 China

**Keywords:** CN-Pt, Hydrogel, Recurrence prevention, Lung cancer, Antitumor immunity

## Abstract

**Background:**

Cancer recurrence following surgical resection is a major cause of treatment failure. Finding effective methods to prevent postoperative recurrence and wound infection is an important component of successful surgery. With the development of new nanotechnology, more treatment options have been provided for postoperative adjuvant therapy. This study presents an innovative hydrogel system that stimulates tumoricidal immunity after surgical resection of non-small cell lung cancer (NSCLC) and prevents cancer relapse.

**Results:**

The hydrogel system is based on the excellent photothermal conversion performance of single-atom platinum (CN-Pt) along with the delivery and release of the chemotherapy drug, gemcitabine (GEM). The system is coated onto the wound surface after tumor removal with subsequent near-infrared (NIR) photothermal therapy, which efficiently induces necroptosis of residual cancer cells, amplifies the levels of damage-associated molecular patterns (DAMPs), and increases the number of M1 macrophages. The significantly higher levels of phagocytic macrophages enhance tumor immunogenicity and sensitize cancer cells to CD8 + T-cell immunity to control postoperative recurrence, which has been verified using an animal model of postoperative lung cancer recurrence. The CN-Pt-GEM-hydrogel with NIR can also inhibit postoperative wound infection.

**Conclusions:**

These findings introduce an alternative strategy for supplementing antitumor immunity in patients undergoing resection of NSCLC tumors. The CN-Pt-GEM-hydrogel with the NIR system also exhibits good biosafety and may be adaptable for clinical application in relation to tumor resection surgery, wound tissue filling, infection prevention, and recurrence prevention.

## Introduction

According to the 2020 Global Cancer Statistics, lung cancer remains the most common type of cancer and the leading cause of cancer-related death [1]. Early-stage non-small cell lung cancer (NSCLC) stages I and II, and sometimes IIIA accounts for one-third of NSCLC, with patients able to achieve good therapeutic results with surgery [2]. However, even patients who are completely resected are at high risk of surgical recurrence and mortality [3]. Therefore, preventing recurrence and metastasis after surgery is the key to ensuring successful outcomes after surgical treatment [4]. With the advancement of medical treatment, techniques such as chemoradiation for patients who undergo surgical tumor removal have been widely applied [5] and additional treatment that incorporates targeted therapies and immunotherapy has also been rapidly developed [6]. 

Several articles have reported that single-atom nanozymes (SAN) exhibit excellent photothermal effects [7] and successively efficient photothermal conversion properties [8], heralding a new era of atomic nanotherapy. Various types of SAN have been used in nanomedicine tumor therapy and in conjunction with multiple therapeutic approaches, such as photothermal therapy (PTT), have demonstrated remarkable efficacy [9]. PTT has gradually attracted attention in the field as a new anti-cancer treatment that is highly effective and non-invasive [10]. A recent study reported that PTT treatments had synergistic antimicrobial effects [11]. Near-infrared (NIR) emerged as a new PTT modality that recently received widespread attention due to its strong tissue penetration, good photostability, and enhanced antimicrobial activity [12]. However, low photothermal conversion efficiency and unsatisfactory therapeutic effects are the main challenges of NIR-PTT [13], and the use of SAN could successfully solve these problems.

Local exposure to NIR light leads to cell swelling and rupture, resulting in speedy and highly specific immunogenic cell death (ICD) of cancer cells [14]. Necroptosis, one of the newly identified forms of ICD [15], is negatively regulated by caspase-8 and dependent on the kinase activity of receptor-interacting protein kinase 1 (RIPK1) and RIPK3 [16]. This phosphorylation event leads to the formation of a pore complex of mixed lineage kinase domain-like protein (MLKL) on the plasma membrane that increases the immunogenicity of tumor cells and results in the secretion of damage-associated molecular patterns (DAMPs) [17], which include surface-exposed calreticulin (CRT), secreted adenosine triphosphate (ATP), and high mobility group protein B1 (HMGB1) [18]. These findings provide new targets and hypotheses for the development of tumor immunotherapy [19]. Necroptosis also induces robust immune responses by releasing DAMPs and various immunoregulatory cytokines [20]. A number of cytokines cause macrophage polarization [21] and the occurrence of specific anti-tumor immunity mediated by T cells [22], promoting changes in the tumor microenvironment.

**Scheme 1:**
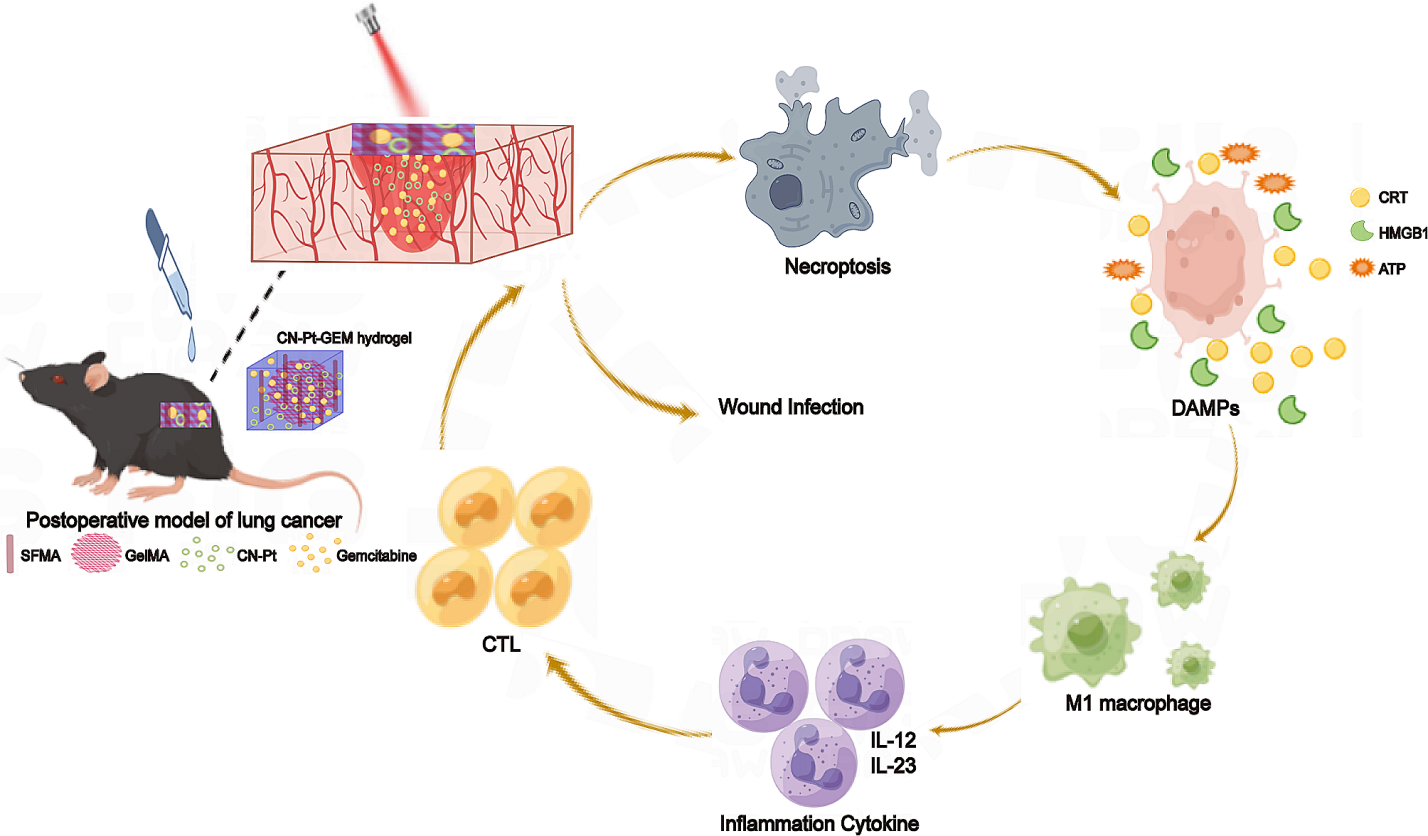
Schematic of CN-Pt-GEM hydrogel system for tumor surgical adjuvant treatment: inhibiting tumor recurrence and wound infection

In this study, we designed and used an innovative strategy based on a combination of single-atom platinum (CN-Pt) and gemcitabine (GEM) with a hydrogel as a medium to deliver and release drugs. While hydrogels have been used topically to promote tissue repair and inhibit wound infection, they can also act as carriers that promote the sustained release of drugs and can be widely used in drug-loading systems. In this study, the CN-Pt-GEM-hydrogel system was used with NIR irradiation on the postoperative surface after tumor removal. This treatment exhibited excellent photothermal conversion performance and highly effective drug-release capacity, inducing necroptosis and immunotherapeutic responses that inhibit local recurrence and postoperative wound infection in NSCLC (Scheme 1).

## Experimental section

### Cell culture conditions

The cell lines used in this study included LLC, A549, HFL1, Human Umbilical Vein Endothelial Cell (HUVEC), MH-S, and RAW264.7. These cells were cultivated in Dulbecco’s Modified Eagle’s Medium (DMEM) supplemented with 10% fetal bovine serum and 1% penicillin and streptomycin, in a 5% CO_2_ environment at 37 °C.

### Animals

C57BL/6 female mice were procured from Guangdong Medical Laboratory Animal Center. All procedural animal methodologies followed the guidelines set by the Guangzhou Medical University Care and Use of Lab Animals, and the experiments received approval from the School of Guangzhou Medical University’s Animal Ethics Committee.

### Preparation of CN-Pt

Preparation of the nitrogen-carbon material included the direct carbonization pyrolysis of chitosan. First, 1 g chitosan powder was placed in a quartz boat and positioned in the plasma enhanced chemical vapor deposition (PECVD) system. Then, all the air was removed from the internal tube of the PECVD system to create a vacuum before inert Argon (Ar2) gas was continuously introduced into the tube at a flow rate of 50 ml/min. Then ramp up the temperature was conducted at a rate of 10 °C/min until it reached 600 °C and a growth time of 4 h and a stop time for insulation was set. Finally, the nitrogen-carbon material was collected from the quartz boat and transferred to a reagent bottle. Then it was stored in a super clean glove box.

Preparation of the CN-600 two-dimensional nitrogen-carbon material. We added 100 mg nitrogen-carbon material powder to ultra-pure water. Using an ultrasonic crusher with a power of 200 W and a switch cycle of 2/2 s, material delamination was conducted for 8 h, after which the undelaminated large nitrogen-carbon material was separated to obtain CN-600 of different sizes. Then, cascade centrifugation was performed on the water dispersion solution to isolate that which contained large material blocks. This solution was then divided equally into two 50 ml centrifuge tubes and centrifuge at 2000 rpm for 10 min. The supernatant of the solution contained the large-sized CN-600 without block bodies. Sequential centrifugation of the supernatant at 4000, 8000, and 12,000 rpm for 10 min each, with the supernatant collected after each centrifugation used for the next round of centrifugation. The precipitate produced from each step included CN-600 nanosheets with gradually decreasing lateral dimensions.

To prepare the CN-600-PEG to improve the stability and biocompatibility of the CN-600 water dispersion, we performed a coating modification with DSPE-PEG-NH_2_. First, 20 mg CN-600 powder obtained by centrifugation at 8000 rpm was added to 10 mL ultrapure water. Then, a certain amount of DSPE-PEG-NH_2_ was added with a feed ratio of 1:5 (mCN-600:mDSPE-PEG-NH_2_ = 1:5). Next, the CN-600 and DSPE-PEG-NH_2_ mixture was placed on a magnetic stirrer at a constant temperature and stirred with a magnetic stir bar for 12 h. The mixture solution was centrifuged at 12,000 rpm for 10 min and the supernatant containing the dispersed DSPE-PEG-NH2 was collected while retaining the precipitate. The obtained precipitate was dried in a vacuum drying oven to obtain CN-600-PEG.

To prepare the metal precursor, 1 g hexahydrated chloroplatinic acid (H_2_Cl_6_Pt·xH_2_O) was added to a mixed solution of 25 mL ultrapure water and hydrochloric acid (ultrapure water: HCl = 24:1 (v/v) with 1 mol/L HCl). The mixture was stirred at constant temperature for 12 h using a magnetic stirrer to obtain a uniformly dispersed metal (Pt) precursor solution (H_2_Cl_6_Pt aqueous solution).

To synthesize CN-600-PEG-Pt, 20 mg CN-600 powder obtained by centrifugation at 8000 rpm was added into 10 mL ultrapure water. A certain amount of uniformly dispersed H_2_Cl_6_Pt aqueous solution was added to achieve a mass ratio of metal precursor to CN-600 of 2%. After the solution was prepared, it stirred at a constant temperature for 12 h using a magnetic stirrer. The mixture solution was centrifuged to remove the unloaded discrete platinum particles. The mixture solution was centrifuged at 12,000 rpm for 10 min and the supernatant was collected while retaining the precipitate. The precipitate was dried using a vacuum drying oven to obtain CN-600-PEG-Pt.

### Transmission electron microscopy

The prepared CN-600-PEG and CN-600-PEG-Pt were dispersed in pure water. Then, 20 µL of the sample solution was dropped onto a carbon-coated copper grid. After complete drying, the morphology of CN-600-PEG and CN-600-PEG-Pt was observed using TEM.

### The particle size and zeta potential

Solutions of CN-600-PEG and CN-600-PEG-Pt were prepared at a concentration of 0.5 mg/mL for use in a laser nanoparticle size analyzer to measure the particle size and zeta potential at a temperature of 25 °C. The measurements were recorded from three parallel experiments for each sample.

### X-ray photoelectron spectroscopy

Ten milligrams of CN-600-PEG and CN-600-PEG-Pt were evenly spread on the sample test bench and the composition of the material was characterized by x-ray photoelectron spectroscopy (XPS), with the test elements being C, N, and Pt.

### Preparation of SFMA

The degummed SF was obtained by immersing 10 g cocoon in 1 L 0.05 M NA_2_CO_3_ solution and boiling it at 100 °C for 30 min, followed by several washes with distilled water. The degummed SF was then dried in an oven for 36 h. To obtain SFMA, 10 g degummed SF was dissolved in 9.3 M LiBr solution at 60 °C for 1 h. The mixture was stirred using a magnetic stirrer and then slowly dripped into 6 mL glycidyl methacrylic acid. After 8 h, the solution was passed through a filter cloth to remove the salt and dialyzed for 7 d using a dialysis bag and distilled water. The obtained SFMA solution was frozen at -80 °C for 12 h and then freeze-dried for 36 h to obtain spongy SFMA.

### Preparation of GelMA

Gelatin was dissolved in 250 mL distilled water at 60 °C before 12 mL methacrylic anhydride was added and the reaction was incubated for 8 h at room temperature, followed by dialysis with distilled water for 3–5 days (retention molecular weight: 3500). The dialyzed solution was decolorized with activated carbon and centrifuged at 8000 rpm before it was passed through a neutral filter paper for lyophilization at -80 °C to obtain GelMA.

### Gel permeation chromatography

A 5 mg aliquot of SFMA and GelMA each were dissolved in pure water and then filtered through a 0.22-µm strainer before being injected it into the GPC system to test the molecular weight fraction of the sample.

### Preparation of hydrogels

In the process of formulating composite hydrogels, the GelMA concentration was consistently maintained at 6% (w/v) in PBS, which was combined with varying quantities of SFMA (2% (w/v), 4% (w/v), and 6% (w/v)) in PBS and mixed into the GelMA solution at a volume ratio of 1:1. Subsequently, 0.1% (w/v) of the photo-initiator, LAP, was added and the solution was irradiated with 405 nm UV light for 10 s to obtain the GelMA/2% SFMA, GelMA/4% SFMA, and GelMA/6% SFMA hydrogels. Similarly, using this preparation method, a GelMA/4% SFMA solution was obtained and then add the desired concentration of Gemcitabine solution and a final concentration of 300 µg/mL CN-Pt solution with 0.1% (w/v) LAP and 405 nm UV light irradiation was used to obtain Hydrogel/CN-Pt and Hydrogel/CN-Pt/Gem.

### ^1^H NMR test

Deuterium was used instead of heavy water as the solvent to dissolve the SF, SFMA, Gel, and GelMA samples. The samples were added to a clean MRI tube after they were completely dissolved and clarified, and then their chemical structure was characterized using ^1^H NMR (Inova-500 M Varian company, America) at room temperature. The atlas was analyzed using MestReNova software.

### Scanning electron microscopy

For SEM, 400 µL of the hydrogels (GelMA, GelMA/2% SFMA, GelMA/4% SFMA, GelMA/6% SFMA, and GelMA/4%SFMA/CN-Pt) were stored in a refrigerator at -80 °C for one night, dried, and surface-coated with gold for 30 s. The surface morphologies of the hydrogels were observed using SEM (S-3400, Hitachi, Japan) with a 5-kV electron beam.

### Swelling rate test

The swelling rate test of the hydrogels was observed using a gravimetric method. In this test, 400 µL of each hydrogel (GelMA, GelMA/2% SFMA, GelMA/4% SFMA, GelMA/6% SFMA, and GelMA/4% SFMA/CN-Pt) were placed in a water bath at 37 °C for 15 min and then demolded. After measuring the dry weight (W_dry_) of the hydrogel, it was placed in PBS (pH 7.4) at 37 °C. The swollen hydrogels were removed from the PBS and weighed (W_swollen_) at predetermined time points after removing excess water using a filter paper. The water absorption ratio (Q) of the hydrogels was calculated as follows: Q = (W_swollen_- W_dry_)/W_dry_ × 100%.

### Rheological test

The rheological behavior of the hydrogels was determined using a 25-mm-diameter stainless steel parallel plate rotary head. The samples (GelMA, GelMA/2% SFMA, GelMA/4% SFMA, GelMA/6% SFMA, and GelMA/4% SFMA/CN-Pt) were scanned by the rheometer (Kinexus, UK Malvern) at room temperature from 0.1 to 10 rad/s to determine the linear viscoelasticity range of the hydrogels and measure the storage modulus (elastic modulus, G′) and loss modulus (viscous modulus, G″). The curves of G′ and G″ were recorded.

### Compression test

Compression tests were evaluated using a universal testing machine (Dynamic universal testing machine ELF3200; Dr. America). In these tests, 600 µL of the hydrogels GelMA, GelMA/2% SFMA, GelMA/4% SFMA, GelMA/6% SFMA, and GelMA/4% SFMA/CN-Pt) were placed under the gel probe, which squeezed the gel until it broke. The force required to break the hydrogel was recorded and defined as the strength of the hydrogel.

### Preparation of the GEM standard curve

Two milligrams of precisely weighed GEM was added to a 10 mL bottle and dissolved in methanol to obtain the GEM stock solution, which was then diluted with methanol to concentrations of 1 µg/mL, 5 µg/mL, 10 µg/mL, 50 µg/mL, and 100 µg/mL. GEM detection was performed using an ultraviolet spectrophotometer at 175 nm. The optical density (OD) value was used as the ordinate, sample concentration was used as the abscissa, and a standard curve was prepared.

### In vitro GEM release rates

For the in vitro release studies, 600 µL of each hydrogel were placed in 2 mL PBS (pH = 7.4) solution and incubated at 37 °C. A sample of the supernatant was collected at each time point and replaced with the same volume of fresh PBS. The collected PBS supernatants were tested for GEM using a photometer to calculate the cumulative release rate.

### Influence of hydrogel concentration on the photothermal effects of the materials

A cylindrical hydrogel was prepared and inserted into a thermocouple thermometer at room temperature. The hydrogel was irradiated with 808-nm NIR (power, 1.5 W/cm^2^) for 5 min and the temperature was recorded at 10 s intervals. Using time point was used as the abscissa and temperature values as the ordinate. The photothermal effects of the hydrogels with different concentrations of CN-Pt were compared in vitro. A blank hydrogel was used as the control.

### Influence of laser power on the photothermal effects of the materials

The liquid surface was irradiated with NIR at different powers (0.5, 1, 1.5, and 2 W/cm^2^) at 808 nm for 5 min. A thermocouple thermometer was inserted into the hydrogel and the temperature was recorded every 10 s at room temperature. A blank hydrogel was used as the control.

### Photothermal stability of the materials

The hydrogel was irradiated with 808-nm NIR (power, 1.5 W/cm^2^) for 5 min and the irradiation was stopped to reduce the temperature to the initial temperature. The experiment was repeated five times. A thermocouple thermometer was inserted into the hydrogel and the temperature was recorded at 10 s intervals at room temperature.

### Cytotoxicity assay

The cytotoxicity of the hydrogel system was evaluated with the CCK-8 assay. LLC, A549, HFL1, and HUVECs cells were cultured in 96-well plates (5 × 10^3^ cells/well) for 12 h before different hydrogels were introduced to the cells. After incubation for 24, 48, or 72 h, the medium was removed and CCK-8 solution diluted in fresh medium was added. After 2 h of incubation, the absorbance was measured at 450 nm using a microplate reader (K3 Plus, BIODL) and cell viability was calculated. To further verify the CCK-8 results, a live/dead staining assay was carried out using calcein and propidium iodide as described earlier, and the cells were visualized using a fluorescence inverted microscope (Zeiss, Germany).

### Apoptosis assay

Cell apoptosis was detected using the Annexin V 633 apoptosis detection kit. The cells were gathered and stained with Annexin V/PI for 30 min at room temperature. The samples were then analyzed using flow cytometry (BD, United States).

### Western blotting analysis

Cells were subjected to lysis by RIPA lysis buffer containing protease and phosphatase inhibitors. Protein extracts were resolved with 10% sodium dodecyl sulfate-polyacrylamide gel electrophoresis and transferred onto polyvinylidene difluoride membranes (Millipore). The membranes were blocked with 5% bovine serum albumin for 2 h at room temperature and then incubated with primary antibodies against GAPDH (Proteintech; 60004-1-Ig, 1:10000), MLKL (Abclonal; A13451), p-MLKL (Abclonal; AP0949, 1:1000; Boster, P00535, 1:1000), RIPK1 (Abclonal; A7414, 1:1000), p-RIPK1 (Abclonal; AP1115, 1:1000), RIPK3 (Abclonal; A5431, 1:1000), and p-RIPK3 (Abcam; ab209384, 1:2000; Abcam, ab222320, 1:1000) overnight at 4 °C. The membranes were then washed with PBS with Tween-20 (PBST) and incubated with secondary antibodies slowly. Using enhanced chemiluminescence (ECL) (Biosharp), the signals were detected using a Tanon infrared imaging system (Guangzhou EWELL Bio-Technology Co., LTD., China).

### DAMPs

A549 cells were treated with different treatment for 24 h. The cells were subsequently rinsed three times with ice-cold PBS and then incubated with anti-CRT antibodies (Proteintech, CL650-27298, 1:200) at 4 °C for 30 min before conducting flow cytometry (BD, United States). The supernatants of the treated A549 cells were also for HMGB1 detection using an enzyme-linked immunosorbent assay (ELISA) kit (MM-13713H1; MEIMIAN). Detection of extracellular ATP release was conducted using an ATP assay kit (S0026; Beyotime).

### qRT–PCR

Total RNA was extracted using AG RNAex Pro Reagent AG21102 [Accurate Biotechnology (Human) Co., Ltd]. RNA was reverse transcribed into cDNA using the Evo M-MLV RT Premix for qPCR AG11706 (Accurate Biotechnology (Human) Co., Ltd). The cDNA was processed in an Applied SYBR Sequence Detection System (LightCycler 480 II, Roche) using a real-time quantitative PCR (SYBR Green, Invitrogen). The primer sequences were as follows:

M-GAPDH-F: CCACCCCAGCAAGGAGAC.

M-GAPDH-R: GAAATTGTGAGGGAGATGCT.

M-IL-12β-F: GGAGACCCTGCCCATTGAACT.

M-IL-12β-R: CAACGTTGCATCCTAGGATCG.

M-IL-23-F: TGGAGCAACTTCACACCTCC.

M-IL-23-R: GGGCAGCTATGGCCAAAAAG.

M-CD-86-F: ATGGACCCCAGATGCACCAT.

M-CD-86-R: TAGGTTTCGGGTGACCTTGC.

### Phagocytosis assay

RAW264.7 LLC cells were treated with Dio and Dil, respectively, for 15 min. The RAW264.7 cells were incubated with the same number of LLC cells in the different treatment groups for 4 h at 37 °C. Phagocytosis was stopped by washing with 4 °C PBS and centrifugation at 1000 rpm/min. The cells were detected using flow cytometry (BD, United States).

### **The animal model for anti-tumor** efficacy

To investigate the therapeutic potential of the hydrogel system, 1 × 10^6^ Luc-LLC cells were injected into the backs with C57BL/6 mice (6–8 weeks of age). After 12 d, mice were randomly divided into six groups (*n* = 6), anesthetized, and approximately 99% of the tumors were surgically removed with sterilized instruments to leave approximately 1% residual tumor to mimic residual micro-tumors post-surgery. Immediately after surgery, various hydrogel treatments, including PBS, hydrogel, hydrogel/CN-Pt, hydrogel/CN-Pt + NIR, hydrogel/Gem, and hydrogel/CN-Pt/Gem + NIR, were applied and followed by the addition of 0.1% LAP and 405 nm UV light exposure for 10 s. The surgical tumor site was then closed by suturing. The tumor size was gauged and calculated using the formula: width^2^ × length × 0.5. The tumors were also evaluated with bioluminescence imaging monitoring. At a dose of 10 µL/g, D-luciferin (40901ES03, YEASEN) in DPBS (15 mg/mL) were intraperitoneally injected into each mouse. The mice were imaged using an IVIS imaging system for 3 min. Animals displaying signs of ill health or bearing tumors exceeding a size of 1.5 cm^3^ were euthanized.

### Flow cytometry

The treated mice from each group were euthanized on day 14. The tumor tissues were dissected into small pieces and digested in 1 mg/mL collagenase IV and 20 µg/mL DNase I in 1640 complete medium at 37 °C for 60 min to obtain cell suspensions. Then, the cells were filtered through a 70 μm nylon cell strainer and used for flow cytometry analysis. The cells were blocked with CD16/CD32 (dilution of 1: 500) to reduce nonspecific antibody binding. For LIVE + CD45 + CD3 + CD4 + and LIVE + CD45 + CD3 + CD8 + T cell analysis, cells were stained with LIVE-APC-H7 (dilution of 1:3000), anti-CD45-PerCP-Cy5.5 (dilution of 1:300), anti-CD3-APC (dilution of 1:300), anti-CD4-BV510 (dilution of 1:300), and anti-CD8-FITC (dilution of 1:300). For LIVE + CD45 + CD11b + F4/80 + CD86 + and LIVE + CD45 + CD11b + F4/80 + CD206 + macrophage analysis, cells were stained with LIVE-APC-H7 (dilution of 1:3000), anti-CD45-PerCP-Cy5.5 (dilution of 1:300), anti-CD11b-FITC (dilution of 1:300), anti-F4/80-BV421 (dilution of 1:300), anti-CD86-PE-CY7 (dilution of 1:300), and anti-CD206-APC (dilution of 1:300). The cells were stained for 30 min at 4 °C and then washed with PBS. After centrifugation and resuspension, the cells were used for flow cytometry (BD, United States).

### In vitro antibacterial experiments

The antibacterial performance of the hydrogels was evaluated using gram-positive *S. aureus* and gram-negative *E. coli*. The concentration of the bacterial suspension was adjusted to 1 × 10^6^ CFU/mL and then 200 µL hydrogel sample were incubated with 100 µL bacterial suspensions. The samples in the GelMA/4% SFMA/CN-Pt group were exposed to NIR laser for 5 min. After treatment, 1.7 mL Luria-Bertani (LB) medium was added to the samples and the cells were cultured at 37 °C for 4 h. An aliquot of 100 µL of the diluted bacterial suspension was used to inoculate LB agar plates, which were cultured for 24 h at 37 °C before the number of culturable colonies was counted. The formula AR (%) = (Nc - Ns)/Nc × 100% was used to calculate the antibacterial rate (AR), where *Nc* is the average bacterial colony number of the control sample and *Ns* is the average bacterial colony number of the hydrogel sample.

### In vivo antibacterial experiments

The rats were treated with 3% sodium pentobarbital sodium (45–60 mg/kg) before the hair around the surgical site was removed with an animal razor and the exposed skin was disinfected with a iodophor. The surgical instruments were sterilized using an autoclaved steam cooker. The skin on the back of the animals was removed using tissue shears. Four infection model wounds with circular skin defects were created on both sides of the back of each rat. The wound diameter was 12 mm and the distance between each wound was approximately 2 cm. The wound was infected with a mixture of 40 µL *S. aureus* (1 × 10^8^ CFU / mL) and *E. coli* (1 × 10^8^ CFU/mL). The medium was added 24 h after infection and the rat was returned to its corresponding cage alone. The change in wound size was recorded every 3 d for 14 d. At predetermined time points, wounds were excised from the mice for the production of paraffin sections, and HE and MT staining of the wound tissues were also performed.

### Statistical analysis

The survival curves were analysed by using the log-rank (Mantel–Cox) test. Differences among multiple groups were evaluated using one-way ANOVA and t-test was used for two-group comparisons. Significant differences are indicated as *P < 0.05, **P < 0.01, ***P < 0.001 and ****P < 0.0001.

## Results

### Structural analysis of CN-Pt

**Fig. 1:**
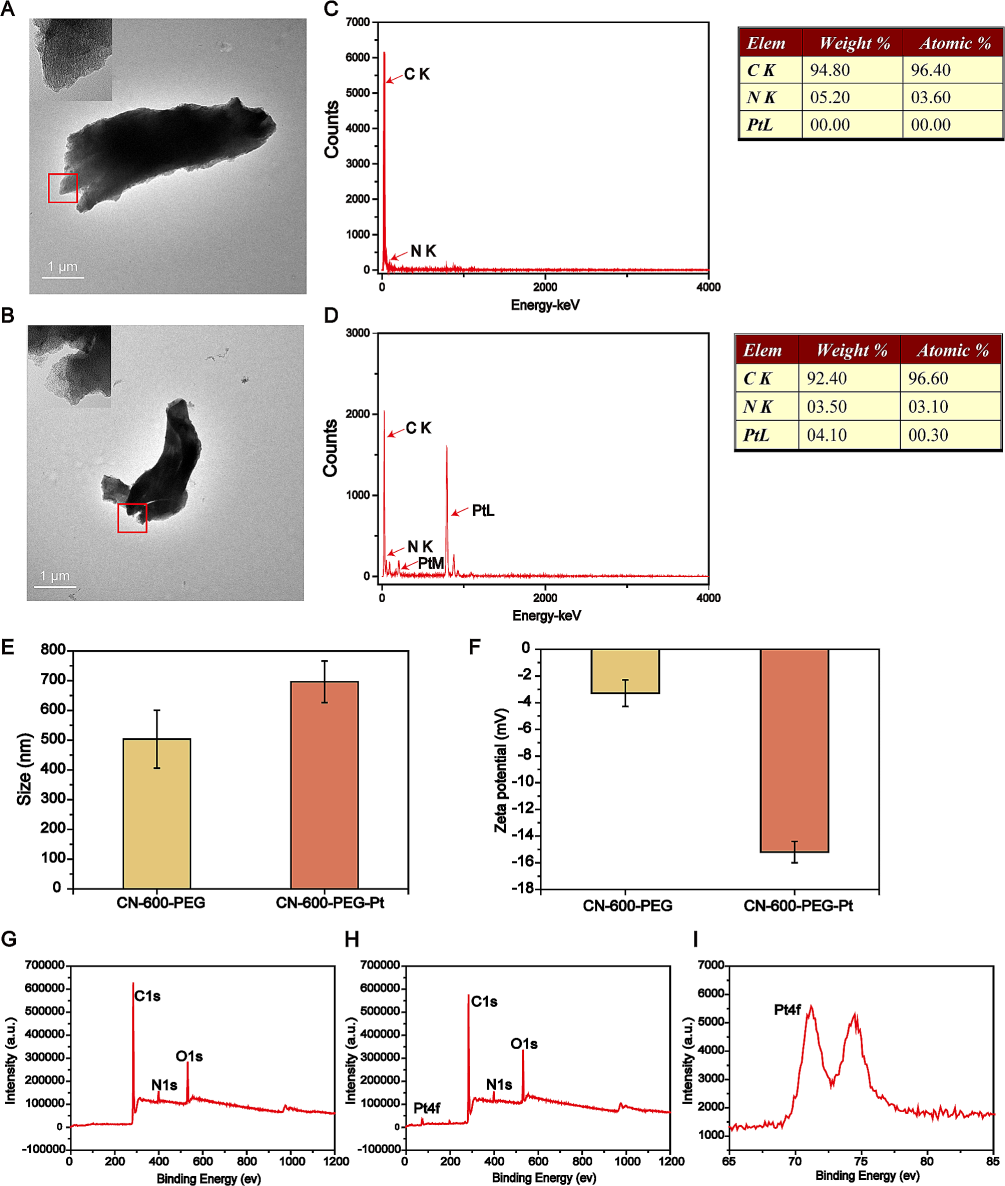
Schematic of CN-Pt. TEM images of (**A**) CN-600-PEG and (**B**) CN-600-PEG-Pt. Elemental analysis of (**C**) CN-600-PEG and (**D**) CN-600-PEG-Pt. (**E**) The particle sizes of each sample. (**F**) Potential diagrams of each sample. The XPS full spectra of (**G**) CN-600-PEG and (**H**) CN-600-PEG-Pt. (**I**) The XPS detailed spectrum of CN-600-PEG-Pt.

The transmission electron microscopy (TEM) imaging and analysis showed that the lateral size distribution of CN-600-PEG and CN-600-PEG-Pt after liquid phase exfoliation was in the range of 1–2 μm (Fig. 1A, B). The few-layer material nanosheets were lighter and relatively smaller compared to the multi-layer aggregates. Local magnification revealed that CN-600-PEG and CN-600-PEG-Pt had a multi-layer structure, with many small particles observed on the surface of CN-600-PEG-Pt, which we hypothesized were reduced Pt atoms. This hypothesis was further supported by elemental analysis (Fig. 1C, D), which confirmed the presence of 0.3% Pt atoms in CN-600-PEG-Pt. We further analyzed the particle size and zeta potential of each sample and found that the hydrated particle size of CN-600-PEG was approximately 500 nm with a potential of -3.29 mV, while the hydrated particle size of CN-600-PEG-Pt was approximately 696 nm with a potential of -15.2 mV (Fig. 1E, F). We further analyzed the composition of the material using x-ray photoelectron spectroscopy (XPS). The XPS full spectrum of CN-600-PEG showed peaks that corresponded to the C, N, and O elements (Fig. 1G). The full and detailed spectra of CN-600-PEG-Pt showed that in addition to the C, N, and O element peaks, the Pt element was also observed (Fig. 1H **and I**). These results mainly describe the preparation of monatomic platinum and its structure.

### Structural analysis and scanning electron microscopy of the hydrogel

**Fig. 2:**
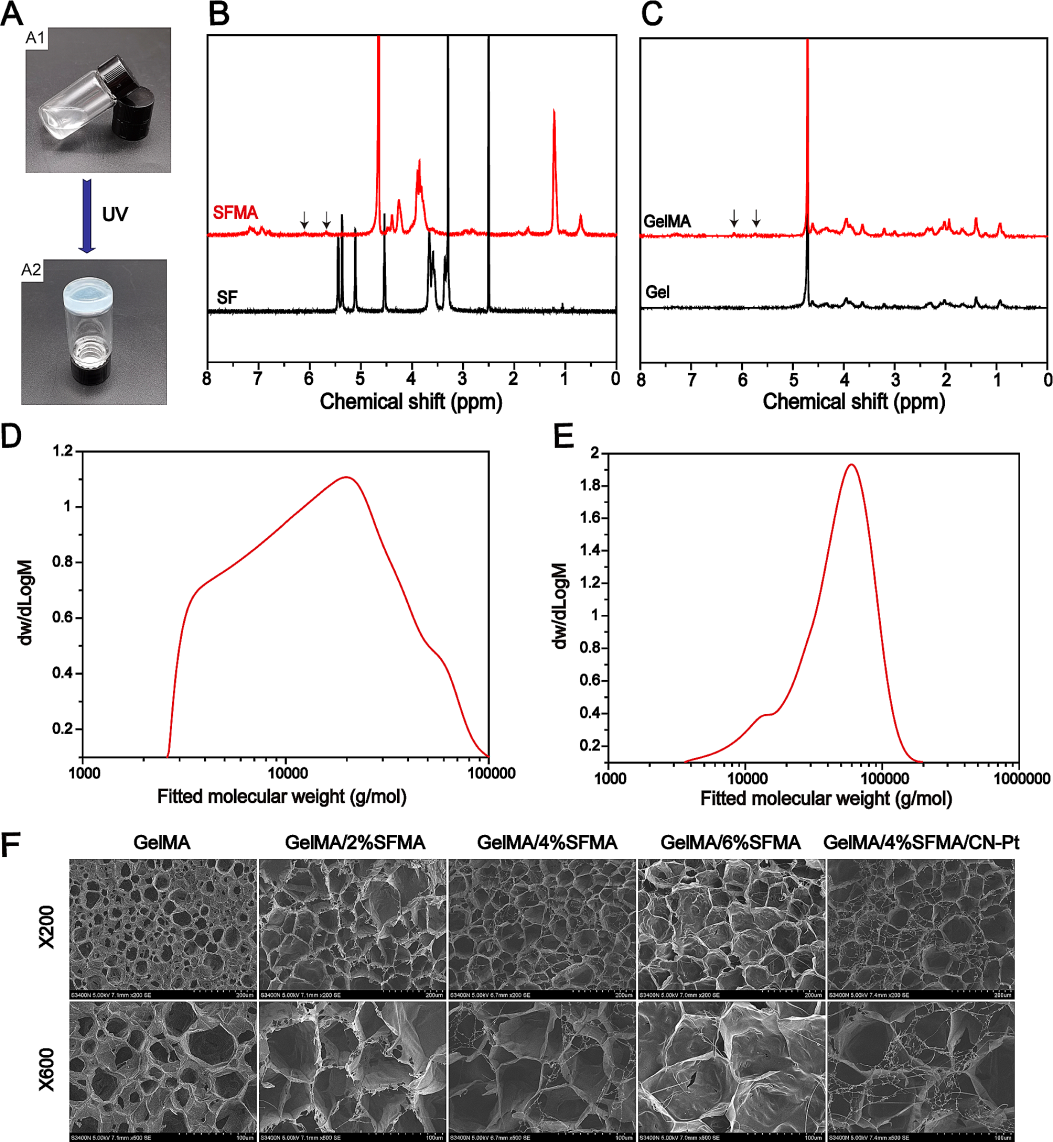
Schematic of the hydrogels. (**A**) Representative photographs of the GelMA/SFMA hydrogel produced by UV-light crosslinking. (**B**) ^1^H-NMR spectra of SF and SFMA. (**C**) ^1^H-NMR spectra of Gel and GelMA. Molecular weight distribution of (**D**) SFMA and (**E**) GelMA. (**F**) SEM images of hydrogels

Methacrylic acid gelatin (GelMA) and methylacrylamide silk fibroin (SFMA) were the two main components of the hydrogels. The GelMA solution was blended with the SFMA solution and in the presence of ultraviolet (UV) light and the photo-initiator lithium phenyl-2,4,6-trimethylbenzoylphosphinate (LAP), free radical polymerization occurs through C = C bonds to form a crosslinking network of GelMA/SFMA hydrogels (Fig. 2A). The ^1^H-nuclear magnetic resonance (NMR) analysis (Fig. 2B) showed that, in comparison with silk fibroin, the hydrogen spectrum of SFMA had new absorption peaks at chemical shifts of 5.6 and 6.1 that corresponded to the absorption peak of hydrogen on methacryloyl, which indicated successful grafting of the methacryloyl group on silk fibroin (SF) and confirmed successful SFMA preparation. Similarly, the hydrogen spectrum of GelMA showed new absorption peaks at chemical shifts of 5.7 and 6.2 in comparison with the gel, which corresponded to the absorption peak of hydrogen on methacryloyl, indicating that the methacryloyl group was successfully grafted on the gel and confirmed the successful preparation of GelMA (Fig. 2C). The gel permeation chromatography (GPC) showed that the average molecular weight of GelMA was 53,037 g/mol, and the average molecular weight of SFMA was 18,866 g/mol (Fig. 2D, E). The morphological features of GelMA and GelMA/SFMA hydrogels after freeze-drying were observed using scanning electron microscopy (SEM; Fig. 2F). The resulting SEM images of the GelMA, GelMA/2% SFMA, GelMA/4% SFMA, GelMA/6% SFMA, and GelMA/4% SFMA/CN-Pt hydrogels showed a three-dimensional porous network structure. In comparison with the pure GelMA hydrogel, the GelMA/SFMA hydrogels had relatively uniform pore sizes.

These results indicated that the addition of SFMA caused the hydrogel to form an interpenetrating network structure that could facilitate the transmission of nutrients and oxygen and maintain a suitable moisture environment. It also could enhance the absorption of wound secretions, which is beneficial for wound healing.

### Cosmetic and physical performance testing

**Fig. 3:**
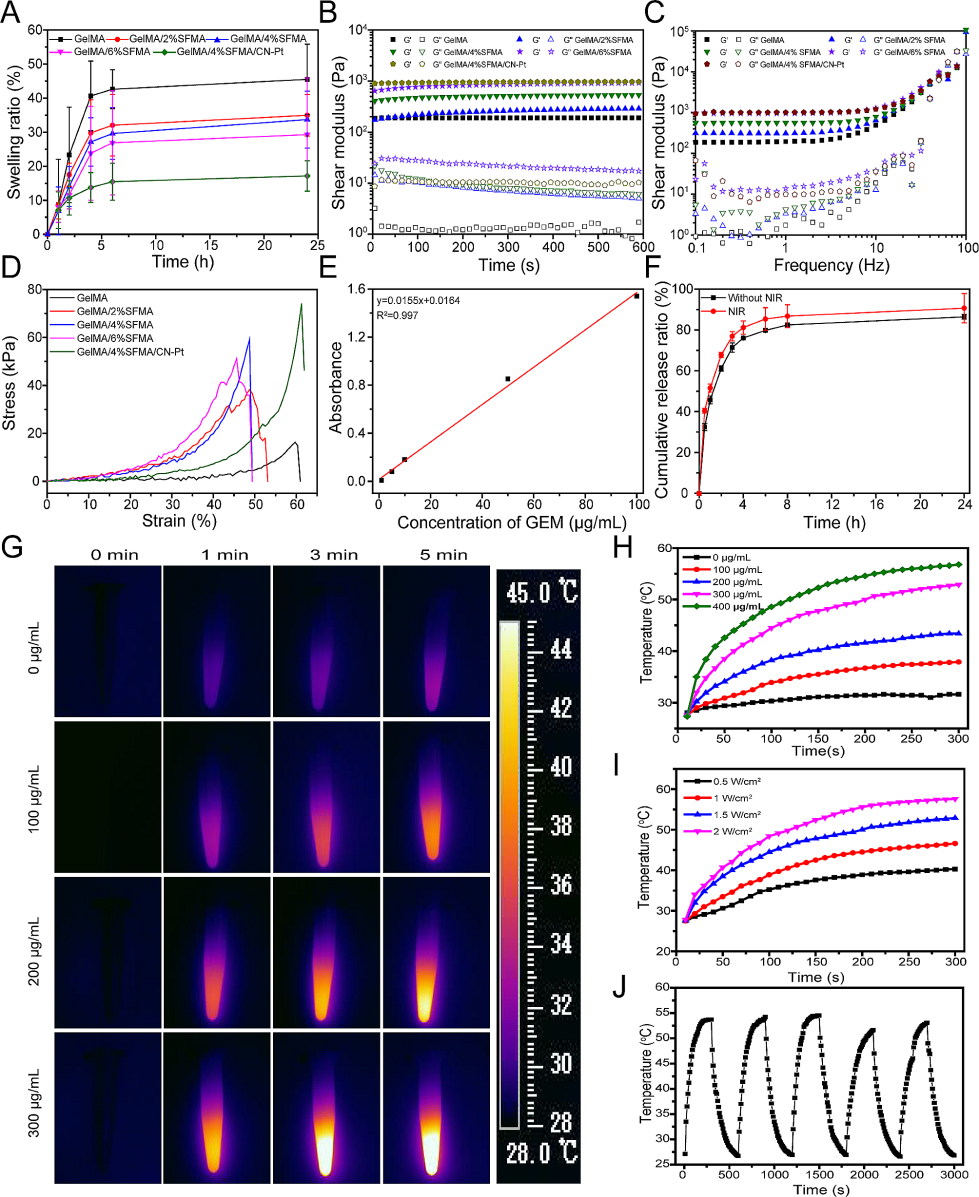
Characterization of the hydrogel system and photothermal conversion performance of CN-Pt. (**A**) The swelling ratio of the hydrogels. (**B**) Variation in the moduli of the hydrogel over time. (**C**) Frequency-dependent analysis of the hydrogel ranging from 0.1 to 100 Hz. (**D**) Compressive curve of the hydrogels. (**E**) Standard curve of gemcitabine. (**F**) Cumulative release curve of gemcitabine. (**G**) Infrared images of hydrogels loaded with different concentrations of CN-Pt. (**H**) Hydrogels loaded with different concentrations of CN-Pt with a power density of 1.5 W/cm^2^. (**I**) Temperature change of the hydrogel over time with irradiation by an 808 nm laser. (**J**) Temperature change plot of hydrogels loaded with 300 µg/mL CN-Pt with a power of 1.5 W/cm^2^ NIR.

All hydrogels reached the swelling equilibrium state after 6 h and a stable swelling rate was maintained, indicating that the hydrogels had excellent dimensional stability (Fig. 3A). The equilibrium swelling rates of the GelMA, GelMA/2% SFMA, GelMA/4% SFMA, GelMA/6% SFMA, and GelMA/4% SFMA/CN-Pt hydrogels were 42.6% ± 5.7%, 32.0% ± 9.0%, 29.6% ± 7.6%, 26.9% ± 15.3%, and 15.5% ± 5.4%, respectively. The higher the SFMA concentration, the lower the swelling rate of the corresponding GelMA/SFMA hydrogels. These results were attributed to the fact that the addition of SFMA supported a higher crosslinking strength for formation and thus, the equilibrium swelling rate of the hydrogel decreased. Good swelling properties favor the absorption of wound exudates by hydrogel dressings. The relationship of G′ (storage modulus) and G″ (loss modulus) with time and frequency of the hydrogel was determined by rheological analysis (Fig. 3B, C). A G′ greater than G″ indicates gel formation. Our results showed that when the hydrogel was gelled, G′ was always greater than G″, indicating that the hydrogel was in a stable gel state as time and frequency increased.

An ideal hydrogel should possess good mechanical properties to maintain its convenience and integrity. The compressive strength of the GelMA hydrogel was 16.2 kPa (Fig. 3D), while the compressive strength of the GelMA/4% SFMA/CN-Pt hydrogel was up to 74.1 kPa. These results showed that the GelMA/4% SFMA/CN-Pt hydrogels exhibited a good degree of crosslinking and had optimal mechanical properties. Moreover, the results also indicated that the compressive strength of the GelMA/SFMA hydrogel was improved by adjusting the concentration of SFMA, highlighting its potential biomedical applications for different tissues and organs. The standard curve of GEM (Fig. 3E) and the drug-release curve (Fig. 3F) of GEM in the presence or absence of NIR showed that the release rate was slightly higher with than without NIR because it accelerated the release of GEM inside the hydrogel. This result confirmed the potential use of hydrogels for in a wide range of drug-loading systems.

### Photothermal Properties

Infrared images obtained with 808-nm laser irradiation indicated that the temperature of the hydrogel system rose rapidly in 5 min and the degree of increase was positively correlated with the material’s concentration (Fig. 3G). The time-temperature curves of the hydrogels at different concentrations were evaluated and showed that the higher the concentration, the more obvious the temperature rise; at a concentration of 300 µg/mL, the temperature rose to 52.9 °C within 5 min (Fig. 3H). We also investigated the photothermal properties of the hydrogels under different power conditions (Fig. 3I). As the power increased, the temperature of the hydrogel and the maximum temperature increased. When the power was 1.5 W/cm^2^, the system temperature rose from 27.6 °C to 52.9 °C, and when the power was 2 W/cm^2^, the system temperature rose from 27.8 °C to 57.6 °C.

We also investigated the photothermal stability of the hydrogels. The hydrogel with a CN-Pt concentration of 300 µg/mL was irradiated with NIR at a power of 1.5 W/cm^2^ for 5 min and then naturally cooled to the starting temperature. This heating and cooling process was repeated five times to record the temperature change curve with time (Fig. 3J). The results showed that the hydrogels had good photothermal stability and that the photothermal conversion efficiency was not affected by five successive temperature-increasing and temperature-decreasing treatments.

### In vitro antitumor efficacy of the hydrogel system and its mechanisms

**Fig. 4:**
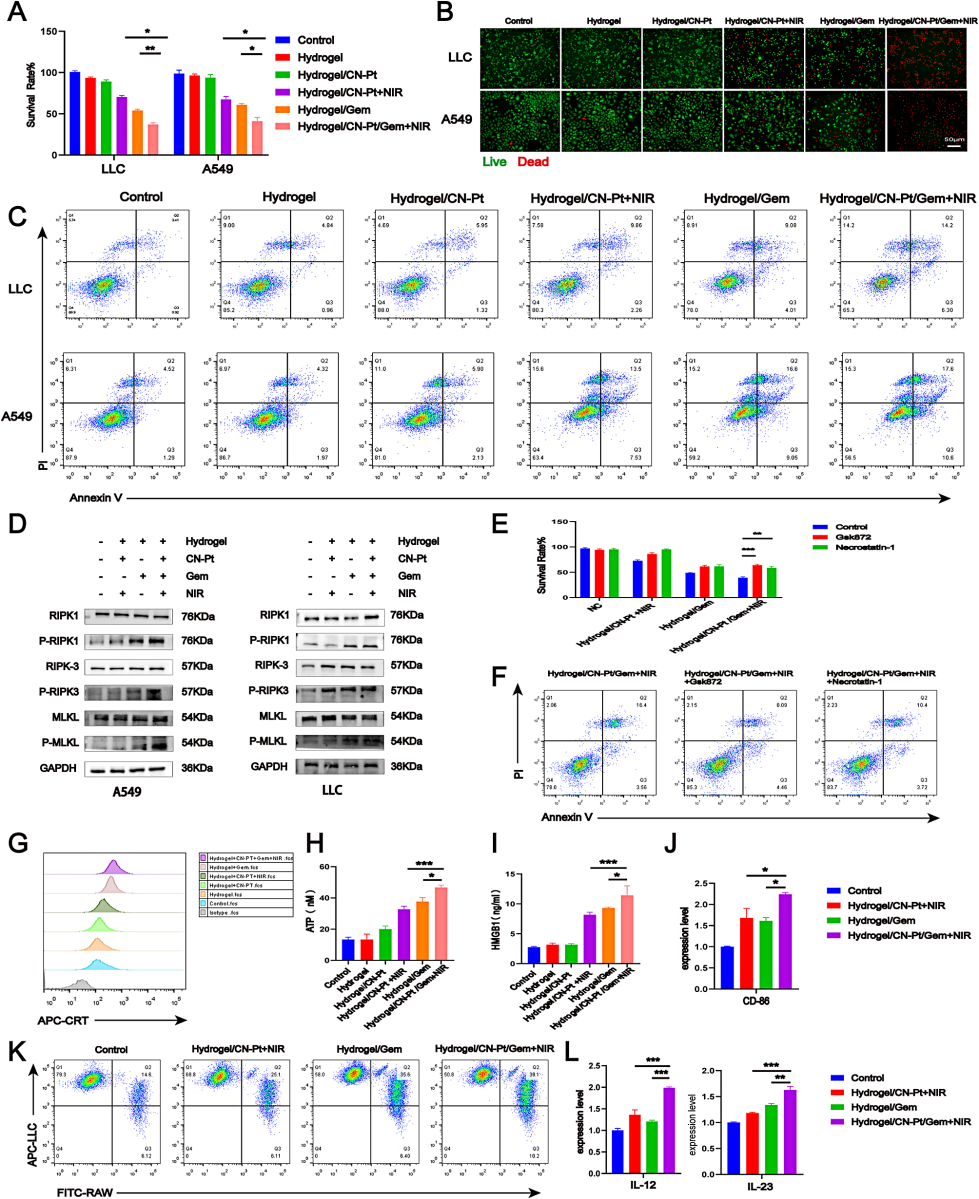
Antitumor effect of NIR-responsive CN-Pt-GEM hydrogel induces necroptosis and immunotherapeutic responses in vitro. The viability of A549 and LLC cells with different treatments were examined using a (**A**) CCK-8 assay and (**B**) live/dead staining assay (live: green, dead: red). (**C**) Flow cytometry quantifying the apoptosis of A549 and LLC cells after exposure to different hydrogel treatments. (**D**) Western blotting of necroptosis marker expression in A549 and LLC cells after different hydrogel treatments. (**E**) CCK-8 assay of the relative viability of LLC cells treated with different hydrogels and necroptosis inhibitors. (**F**) Necroptosis inhibitor treatment decreased the apoptosis of LLC cells in the Hydrogel/CN-Pt/Ge + NIR group. (**G**) CRT exposure at the cell surface of A549 cells treated with different hydrogels was detected by flow cytometry on. (**H**) Extracellular ATP secretion from A549 cells was assessed with an ATP kit. (**I**) The HMGB1 release profile of A549 cells was measured with an ELISA kit. (**J**) qRT-PCR analysis of *CD86* expression in MH-S cells co-cultured with LLC cells treated with different hydrogels. (**K**) Flow cytometry showing the percentage of phagocytic macrophages in a co-culture of RAW264.7 and pre-treated LLC cells. (L) Relative expression of *IL-12* and *IL-23* in MH-S cells co-culture with pre-treated LLC supernatant was measured by qRT-PCR.

To validate the therapeutic effects of the hydrogel system, we examined its antitumor activity on LLC and A549 cells in vitro. The results of the CCK-8 and live/dead staining assays indicated that the hydrogel/CN-Pt/GEM + NIR group displayed the strongest inhibitory effect against LLC and A549 cells among all treated groups (Fig. 4A, B). To further verify the antitumor efficiency, the apoptotic and necrotic effects of the hydrogel were analyzed using flow cytometry, which showed that the necrosis rate was higher in the hydrogel/CN-Pt/GEM + NIR group than in the hydrogel/CN-Pt + NIR or hydrogel/GEM groups (Fig. 4C). These results suggested that the hydrogel system successfully enhanced antitumor effects in these cell lines.

We also explored hydrogel-induced cell death using western blotting (WB) and restoration assays. The levels of phosphorylated necroptosis markers, RIPK1, RIPK3, and mixed-lineage kinase domain-like pseudokinase (MLKL), were significantly increased in the hydrogel/CN-Pt/GEM + NIR group (Fig. 4D). We used necroptosis inhibitors (3 µM GSK-872; 30 µM necrostatin-1) to further confirm the role of necroptosis in the hydrogel system. The necroptosis inhibitors reduced the cell death induced by the hydrogel/CN-Pt + NIR, hydrogel/GEM, and hydrogel/CN-Pt/GEM + NIR in the CCK-8 assays (Fig. 4E), which was consistent with the flow cytometry results from the hydrogel/CN-Pt + NIR group (Fig. 4F). Treatment with hydrogel/CN-Pt/GEM + NIR, which included hydrogel/CN-Pt + NIR and hydrogel/Ge, elicited calreticulin (CRT) exposure in tumor cells when compared to that in the control, hydrogel, and hydrogel/CN-Pt groups (Fig. 4G). Hydrogel/CN-Pt/GEM + NIR-treated tumor cells showed the highest HMGB1 expression and ATP secretion among the tested groups (Fig. 4H, I).

A correlation was observed between the different treatments and macrophage polarization, which was assessed by quantitative reverse transcription-polymerase chain reaction (qRT-PCR). The analysis showed that MH-S cells co-cultured with LLC cells treated with hydrogel/CN-Pt/GEM + NIR exhibited a CD86-high phenotype in comparison with those co-cultured with LLC cells treated with other hydrogels (Fig. 4J). The RAW264.7 cells were stained with Dio and the LLC cells that were pre-treated with the different hydrogels were stained with Dil. After 4 h of co-culture, the RAW264.7 cells phagocytosed the LLC cells (double-positive cells). Furthermore, RAW264.7 cells co-cultured with LLC cells treated with hydrogel/CN-Pt/GEM + NIR showed a significantly higher phagocytic index than those co-cultured with LLC cells treated with the other hydrogels (Fig. 4K). The secretion of cytokines by M1 macrophages, including interleukin (IL)-12 and IL-23, further confirmed the effective immune responses induced by hydrogel/CN-Pt/GEM + NIR (Fig. 4L). These findings demonstrate that hydrogel/CN-Pt/GEM + NIR can induce necroptosis and improve the immunosuppressive tumor microenvironment in vitro.

### The hydrogel system significantly suppressed tumor recurrence and prolonged the survival of postresection NSCLC mouse model

**Fig. 5:**
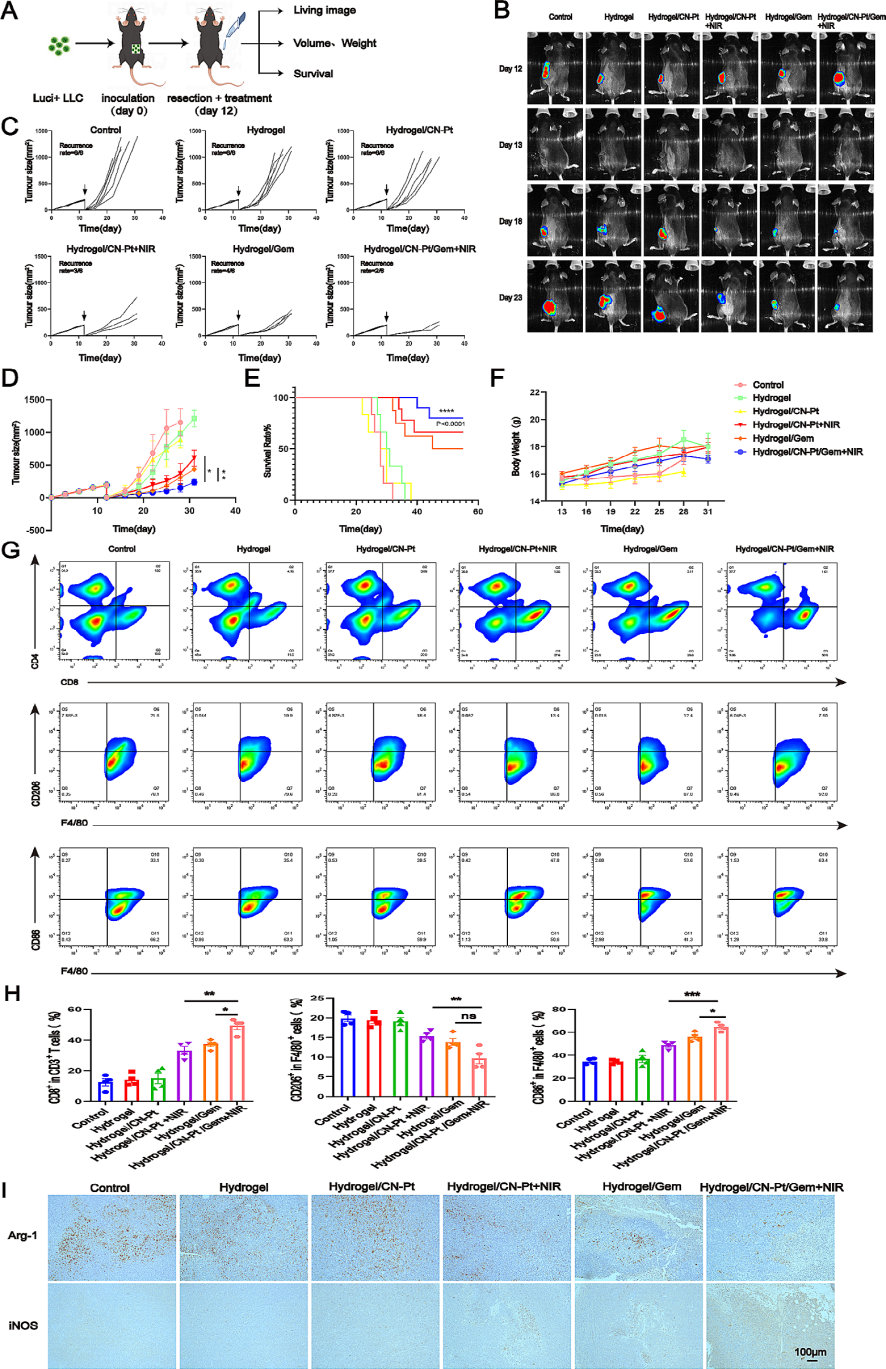
CN-Pt-GEM hydrogels reduce recurrence and induce immunotherapeutic responses in LLC tumors after surgery. (**A**) Schematic of the experimental design of a mouse model for incomplete tumor resection. (**B**) In vivo bioluminescence imaging of LLC tumors after removal of the primary tumor. (**C**) Individual and (**D**) average tumor growth kinetics in the different treatment groups. (*n* = 6 biologically independent animals per group). (**E**) Survival of mice corresponding to the tumor size after different hydrogel treatments. Statistical significance was calculated using the log-rank Mantel–Cox test. (**F**) Weight changes of mice in different treatment groups. (**G**) Representative flow cytometry results and (**H**) quantitative analysis and (**I**) immunohistochemistry of CD3 + CD8 + T cells, M1-like macrophages and M2-like macrophages in postresection LLC-bearing tumor tissue

To gain a global understanding of the influence of the hydrogel system on prevent postoperative recurrence, we used an incomplete tumor resection mouse model. On day 12 after subcutaneous tumorigenesis using Luci + LLC cells in mice, the tumor was surgically removed, leaving ~ 1% residuum as a postoperative NSCLC model. Mice were randomly assigned to different treatment groups (Fig. 5A). In the initial five days after each treatment, more tumor suppression was observed in the hydrogel/CN-Pt/GEM + NIR-treated group than in the other groups (Fig. 5B). Mice treated with hydrogel/CN-Pt/GEM + NIR showed improved control of tumor regrowth, indicated by four out of six mice that had no detectable tumors (Fig. 5C, D). 60% of the mice that received the hydrogel/CN-Pt/GEM + NIR treatment survived for at least 55 days (Fig. 5E), and their body weights were not affected by the treatment (Fig. 5F).

We also explored the effects of the hydrogel system on the tumor immune microenvironment. Residual tumors were harvested and analyzed by flow cytometry and immunohistochemistry staining 14 days after resection and treatment. We observed a reduction in M2 macrophages and an increase in M1 macrophages in tumors treated with hydrogel/CN-Pt/GEM + NIR when compared with the other groups. Remarkably, hydrogel/CN-Pt/GEM + NIR treatment also caused an increase in CD8 + T cells (Fig. 5G, H). Consistent with these results, the immunohistochemical assessments indicated that hydrogel/CN-Pt/GEM + NIR treatment promoted immune responder populations, which is an essential step to mount antitumor immunity (Fig. 5I). Together, these results suggest that the hydrogel system relieved the immunosuppressed tumor microenvironment, had an effective tumoricidal immune function and inhibited lung cancer recurrence after surgery.

### Antimicrobial effects of CN-Pt-GEM hydrogel

**Fig. 6:**
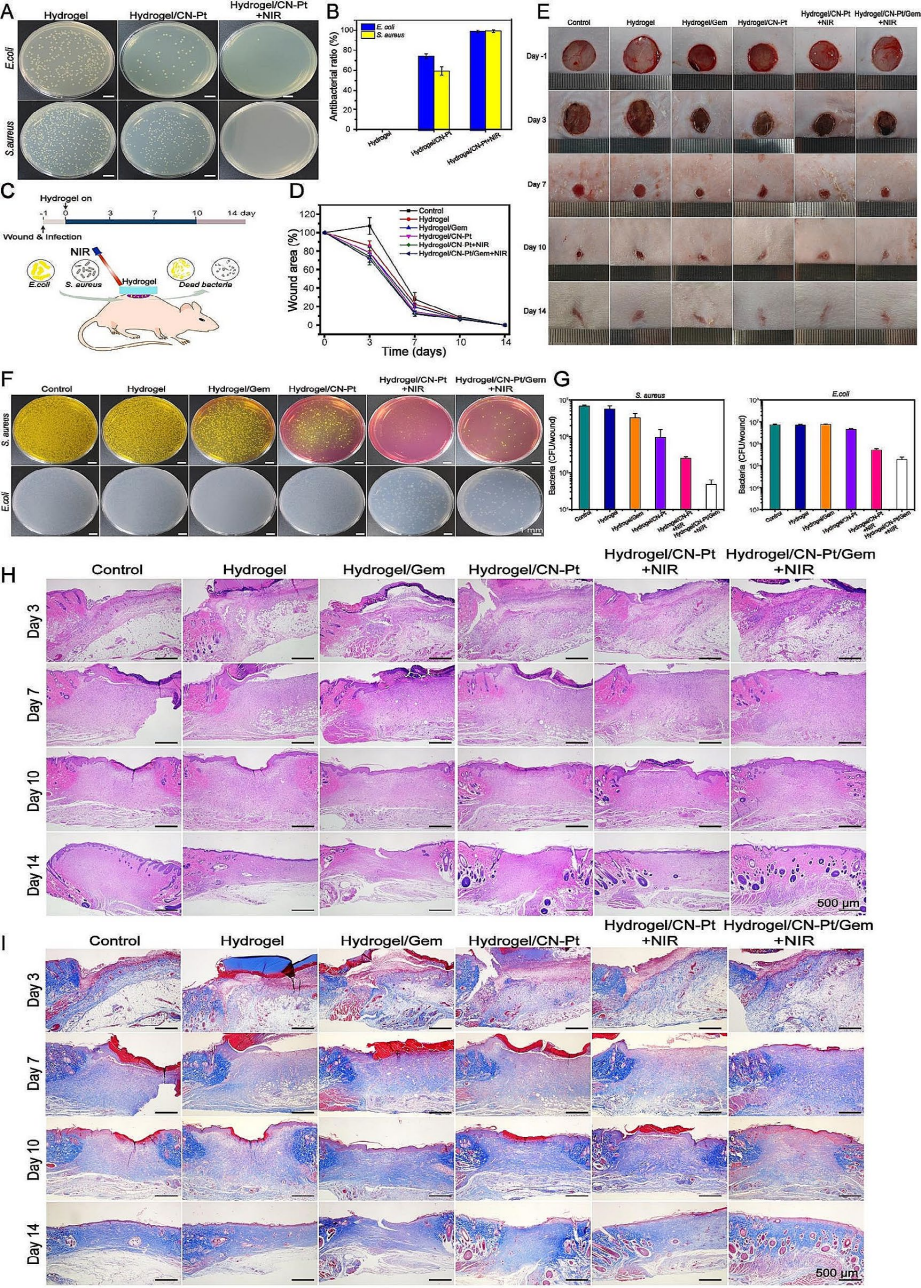
Validation of antibacterial effect and accelerated wound healing of the CN-Pt-GEM hydrogel. (**A**) Images of *S. aureus* and *E. coli* colonies in co-cultured with different treatments. (**B**) *S. aureus* and *E. coli* bacterial: antibactial ratio after different hydrogel treatments. (**C**) Schematic diagram of antimicrobial model in vivo. (**D**) Representative photos of the infectious wounds after different hydrogel treatments. (**E**) Graph of wound size change. (**F**) A map of bacterial growth in the rat wound on the third day. (**G**) Quantitative analysis of *S. aureus* and *E. coli* on the third day. (**H**) Representative H&E staining in wound tissues. (**I**) Representative Masson’s trichrome staining of wound tissues

Postoperative care for lung cancer is an important part of therapeutic surgery, with many cases of postoperative care failure caused by bacterial infection, highlighting the importance of developing biomaterials with bacteriostatic effects for clinical application. Therefore, we evaluated the antibacterial effects of the prepared hydrogels against two representative microorganisms, the gram-positive *Staphylococcus aureus* and gram-negative *Escherichia coli in vitro*. Hydrogel/CN-Pt + NIR showed better antibacterial performance than hydrogel/CN-Pt after 4 h of bacterial exposure at 37 °C, with the hydrogel/CN-Pt + NIR killing almost 100% of *S. aureus* and *E. coli* cells (Fig. 6A, B). These results showed that CN-Pt had some antibacterial activity, which was enhanced with NIR.

We further verified the effect of the hydrogel system on postoperative infection by establishing an animal model of wound infection in rats, and then evaluated the bacterial residue and effect on tissue repair in different treatment groups (Fig. 6C). In the hydrogel treatment group, the wound healing and promoting tissue repair effect was much faster in comparison with the other groups (Fig. 6D, E). Furthermore, to determine bacterial growth in surgical wounds in rats, we homogenized the wound tissue and observed the development of bacteria on the third postoperative day. All results demonstrated that hydrogel/CN-Pt/GEM + NIR had the lowest amount of *S. aureus* and *E. coli*, indicating that the prepared hydrogel could effectively suppress wound infection and enhance wound healing (Fig. 6F, G). To further investigate the effect of hydrogel systems on the regeneration of epidermal and dermal cells, we treated the wound tissue with hematoxylin-eosin (HE) and Masson’s trichrome (MT) staining. More particle tissues were observed in the hydrogel-treated group and overall, the slowest wound healing was shown in the control group (Fig. 6H). Collagen fibers are stained blue with MT staining and can be used to assess collagen deposition at the healing site. In comparison with the other groups, the wounds in the hydrogel/CN-Pt/GEM + NIR group were significantly reduced, showed more orderly collagen deposition, wider gaps between collagen, and regeneration of some hair follicles (Fig. 6I).

### Biological safety evaluation in vitro and in vivo

**Fig. 7:**
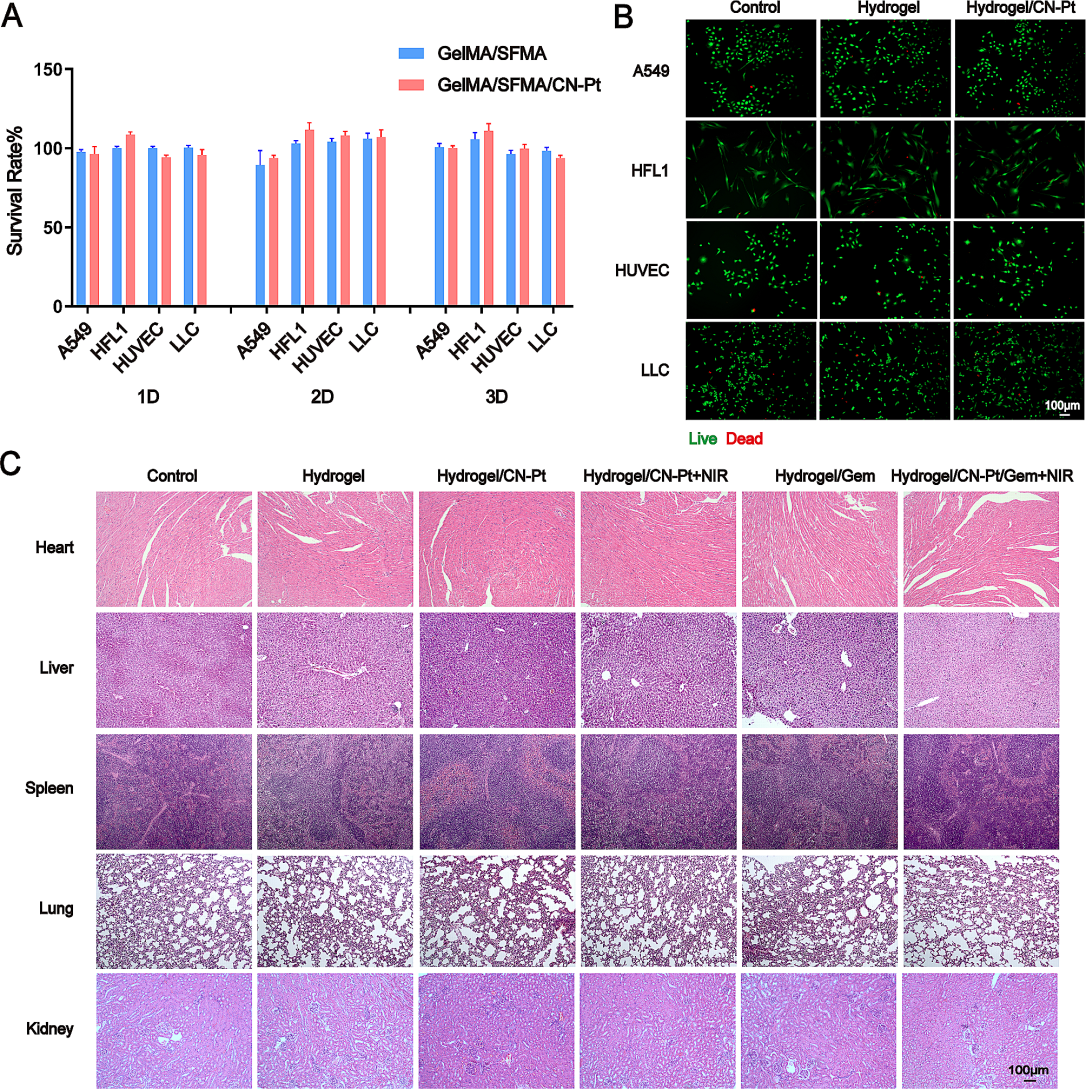
Biological safety evaluation of the hydrogel system in vitro and in vivo. (**A**) Cell viability of A549, HFL1, HUVEC, and LLC cells treated with different hydrogels for three days before examined using a CCK-8 assay. (**B**) Live/dead staining assay of cells in different treated groups (live: green, dead: red). (**C**) Representative H&E staining images of heart, liver, spleen, lung, and kidneys from mice in different hydrogel treatment groups

Nanomaterial biosafety is a major concern in clinical practice since it will potentially affect postoperative discomfort and patient acceptability. In the present study, CCK-8 and live/dead staining assays were used to assess the biosafety of non-drug-loaded hydrogels. We also treated mice with different hydrogels after 14 days and then tested the main organ function to investigate biocompatibility dynamics. The results of the in vitro experiments suggested that after 24, 48, and 72 h, the cell survival rate remained higher than 90% (Fig. 7A). The live/dead staining assay under different treatment also revealed no significant cell death after 24 h of treatment (Fig. 7B). The HE staining analysis of the major organs also showed good biological safety in mice (Fig. 7C). These in vitro and in vivo results demonstrated that the hydrogels exhibited good biological safety and have promising potential clinical applications.

## Discussion

Lung cancer has the highest global incidence and mortality rate of cancer-related diseases [23]. Although patients with early-stage lung cancer have greatly benefited from surgery, these patients are still at high risk of recurrence and death [24]. NIR-PTT has become an important adjuvant therapy in recent years. It can not only kill tumor cells directly through photothermal action, but can also cause tumor cells to expand rapidly, blister and eventually rupture, thus inducing ICD and activating the antitumor immune response, including changes in the tumor microenvironment via activating effect factors and promoting the secretion and release of cytokines [25]. Thus, NIR-PTT has emerged as a promising postoperative therapeutic strategy. However, an important question of whether the photothermal conversion efficiency of NIR-PTT can be improved still remains.

Inspired by highly active SAN catalysis therapy [26], it was confirmed that single atoms can enhance photothermal conversion [27]. In our study, we developed a novel single-atom Pt (CN-Pt) from metal nanoparticles that have excellent photothermal conversion performance and photothermal stability. On the basis of these findings, we designed a hydrogel with CN-Pt and the chemotherapy drug GEM that can be applied with NIR irradiation to the postoperative surface after tumor removal. Although NIR-PTT can induce ICD, the specific role of necroptosis, a form of ICD, in these processes remains unclear. Our study showed that the combination of the CN-Pt-GEM hydrogel and NIR upregulated the expression of necrosomes and receptor-interacting proteins RIP1 and RIP3, which are the principal components of necroptosis [28]. Our experimental results confirmed that the combination of the CN-Pt-GEM hydrogel with NIR induced DAMPs, including surface-exposed calreticulin (CRT), ATP secretion, and release of HMGB1, which is consistent with previously reported findings [29]. The release of DAMPs demonstrates cytokine secretion [30] and this cytokine storm results in macrophage activation [31]. Our experimental results demonstrate that CN-Pt-GEM hydrogels with NIR promoted polarization of macrophages towards to the M1 anti-tumor phenotype, as well as reduced the population of cells exhibiting the M2 pro-tumor phenotype. These changes are beneficial for reactivating the macrophage immune response and improving the tumor immunosuppressive microenvironment [32]. M1 macrophages secrete cytokines such as IL-12 and IL-23 that play roles in antigen presentation, activation of the immune response, and inhibition of tumor progression [33]. The CN-Pt-GEM hydrogel with NIR increased the number of M1 macrophages and the effects of phagocytic tumor cells, consequently enhancing antitumor T-cell immunotherapeutic responses.

Bacterial wound infections are a leading cause of surgical failure and a common postoperative complication. The outstanding biocompatibility and strong adhesion attributes of hydrogels present compelling benefits as a wound dressing [34]. We confirmed the antibacterial effect of the CN-Pt-GEM hydrogel with NIR irradiation and provide evidence for the use of the CN-Pt-GEM hydrogel with NIR as a strategic adjuvant therapy for tumor surgery.

Our results confirmed that the CN-Pt-GEM hydrogel with NIR induces necroptosis and immunotherapeutic responses. However, necroptosis is only one of the death forms of ICD and other studies have found that ICD also includes apoptosis, ferroptosis, pyroptosis, cuproptosis and potentially other modes still undiscovered [35]. Therefore, additional studies are required to verify whether other cell death modalities are involved in this process. Nevertheless, the NIR-responsive CN-Pt-GEM hydrogel induced necroptosis and immunotherapeutic responses that inhibited postoperative recurrence and wound infections in lung carcinoma. However, in order to apply the hydrogel system in clinic translation in the future, more experimental methods and clinical studies are needed to verify its anti-tumor efficacy and biological safety. Larger sample sizes and longer follow-up periods would strengthen the reliability and generalizability of the research results.

## Conclusions

In summary, we developed a novel post-surgical cancer immunotherapy strategy using a CN-Pt-GEM hydrogel at the tumor resection site in combination with NIR irradiation, which induced necroptosis, facilitated reversal of the immunosuppressive tumor microenvironment, and induced systemic immunological responses that inhibited local tumor recurrence. This approach also prevented postoperative wound infections and the CN-Pt-GEM hydrogel system has good biosafety, supporting the potential clinical translation of this method for tumor resection.

## Data Availability

No datasets were generated or analysed during the current study.
